# Polyreactivity of antibodies from different B-cell subpopulations is determined by distinct sequence patterns of variable region

**DOI:** 10.3389/fimmu.2023.1266668

**Published:** 2023-11-23

**Authors:** Maxime Lecerf, Robin V. Lacombe, Jordan D. Dimitrov

**Affiliations:** Centre de Recherche des Cordeliers, INSERM, CNRS, Sorbonne Université, Université Paris Cité, Paris, France

**Keywords:** antibodies, variable regions, antibody polyreactivity, sequence analyses, molecular modeling

## Abstract

An antibody molecule that can bind to multiple distinct antigens is defined as polyreactive. In the present study, we performed statistical analyses to assess sequence correlates of polyreactivity of >600 antibodies cloned from different B-cell types of healthy humans. The data revealed several sequence patterns of variable regions of heavy and light immunoglobulin chains that determine polyreactivity. The most prominent identified patterns were increased number of basic amino acid residues, reduced frequency of acidic residues, increased number of aromatic and hydrophobic residues, and longer length of CDR L1. Importantly, our study revealed that antibodies isolated from different B-cell populations used distinct sequence patterns (or combinations of them) for polyreactive antigen binding. Furthermore, we combined the data from sequence analyses with molecular modeling of selected polyreactive antibodies and demonstrated that human antibodies can use multiple pathways for achieving antigen-binding promiscuity. These data reconcile some contradictions in the literature regarding the determinants of antibody polyreactivity. Moreover, our study demonstrates that the mechanism of polyreactivity of antibodies evolves during immune response and might be tailored to specific functional properties of different B-cell compartments. Finally, these data can be of use for efforts in the development and engineering of therapeutic antibodies.

## Introduction

An antibody (Ab) molecule that can interact with multiple unrelated antigens is referred to as polyreactive (1–3). Polyreactive Abs are normal constituents of immune repertoires and have important functions. For example, they participate in the first line of defense against pathogens (4–6) and contribute to establishing mutualistic equilibrium between the host and microbiome (7–9). Notably, many of broadly neutralizing Abs against HIV-1 and influenza virus display antigen-binding polyreactivity (10–16). Polyreactive antibodies also participate in the clearance of apoptotic cells and damaged macromolecules (17–19). Nevertheless, polyreactivity is considered a negative trait for therapeutic Abs (20). It can cause deterioration of the pharmacokinetics of therapeutic Abs and poses a risk of undesirable effects (20). Moreover, polyreactivity correlates with other liabilities of Abs, such as the tendency for self-association (21, 22).

Since the discovery of polyreactive Abs, research efforts have been dedicated to unraveling its molecular basis. These efforts were boosted following the identification of polyreactivity as a developability risk for therapeutic Abs. Many features of variable (V) domains have been associated with polyreactivity, but controversial results are often reported in the literature. Thus, polyreactive Abs were shown to contain an elevated number of positively charged amino acid residues in their CDR H3 (15, 23–28). The presence of patches of positive charges on the molecular surface of the antigen-binding site or unbalanced distribution of charges has also been associated with polyreactivity of Abs (29–33). Analyses of synthetic Ab libraries revealed that some hydrophobic and aromatic residues in CDR H3 could also contribute to polyreactivity (34). Conversely, a recent study of large repertoires of mouse and human Abs found a tendency for neutrality, or absence of a particular predominance of charges or hydrophobicity, in antigen-binding sites of polyreactive Abs (35). Polyreactivity was also associated with a longer CDR H3 loop (23, 27, 36, 37) and a higher tendency of this loop to form β-sheets (38). Other works, however, failed to detect differences in the size of CDR H3 between polyreactive and monoreactive Abs (8, 15, 24, 35). A global trait of V domains that has been related to promiscuity is conformational dynamics (3, 39). Many studies demonstrated that paratopes of polyreactive Abs have increased conformational flexibility (40–48). Nonetheless, there are reports demonstrating that some Abs can display polyreactivity without substantial conformational dynamics (13, 35, 49).

The conflicting results about the role of specific sequence or molecular attributes of Ab polyreactivity imply that there is still incomplete understanding of how Ab molecules attain promiscuous antigen binding. Several reasons can explain the conflicting results in the literature. They may be the result of the use of different experimental assays for assessment of Ab polyreactivity, the use of different statistical methods for analyses of data, the use of limited sets of Abs, evaluation of polyreactivity of Abs from different species (human vs. mouse), and assessment of polyreactivity of Ab repertoires biased by selection for a particular antigen specificity. They may also reflect the existence of multiple mechanisms of polyreactivity.

Recently, we implemented a sequence analyses approach where the polyreactivity of clinical-stage therapeutic Abs was correlated with the frequency of each amino acid residue, type of residues, and some global features (length of CDRs, number of somatic mutations) of V domains (32, 50, 51). These data revealed specific sequence traits associated with natural or induced by pro-oxidative substances polyreactivity in therapeutic Abs. We anticipated that application of the same approach for analyses of large human Ab repertoires would allow deciphering determinants of polyreactivity.

We used data sets from two recent studies (27, 37) and analyzed the sequence patterns that determine the polyreactivity in human Ab repertoires. Importantly, we applied the analyses to subcategories of Abs identified on the basis of the B-cell type from which they were cloned. We also applied molecular modeling to predict the structure of top polyreactive Abs from each category. Our data revealed several unique sequence patterns determining the polyreactivity of human Abs. Notably, polyreactivity of Abs from different B-cell subpopulations was determined by distinct sequence patterns of V domains. Our study also showed that human Ab might use various molecular mechanisms of polyreactivity. These results might have important repercussions for understanding the mechanism and biological functions of polyreactive Abs.

## Results

### Human monoclonal Abs subjected to analyses

We used the data sets from two studies where monoclonal Abs were cloned from different subpopulations of healthy human B cells. Thus, we analyzed sequences of 398 Abs characterized in the study of Shehata et al. (27). These Abs were cloned from naive, IgM memory, and IgG memory B cells and from long-lived plasma cells (LLPCs). In addition, we evaluated sequence correlates of polyreactivity of 240 monoclonal Abs cloned from IgA memory B cells, described in the study of Prigent et al. (37). In both studies, Abs were cloned without selection for a particular antigenic specificity and expressed exclusively as IgG1 for functional analyses. Of note, the two studies used different approaches for the estimation of Ab polyreactivity. Thus, Shehata et al. used a technique referred to as polyspecificity reagent assay (PSR), where Ab reactivity to proteins available in human cell lysate is measured by flow cytometry and presented as a gradually increasing numeric score (27). Prigent et al. assessed Ab polyreactivity by ELISA (37). To use the semi-quantitative data presented in the work of Prigent et al. in our correlation analyses, we first assigned numeric scores of reactivity based on the number of recognized antigens by a given Ab (see *Methods*).

In previous works, V region sequence patterns that determined polyreactivity were evaluated by using pools of Abs that originate from different B-cell subpopulations. Moreover, some of these studies used Abs directed to pathogens (influenza virus or HIV-1) or Abs designed to be used as therapeutics. Here, we aimed to assess the sequence traits of V_H_ and V_L_ regions that correlate with Ab polyreactivity using Abs without pre-defined antigen specificity. We also considered it important, in addition to bulk analyses, to segregate the Abs as based on their origin (B-cell types from which they were isolated) and perform the statistical analyses individually.

### Correlation analyses of sequence features of V_H_ and V_L_ regions determining the polyreactivity of Abs from different B-cell subpopulations

**Figure 1:**
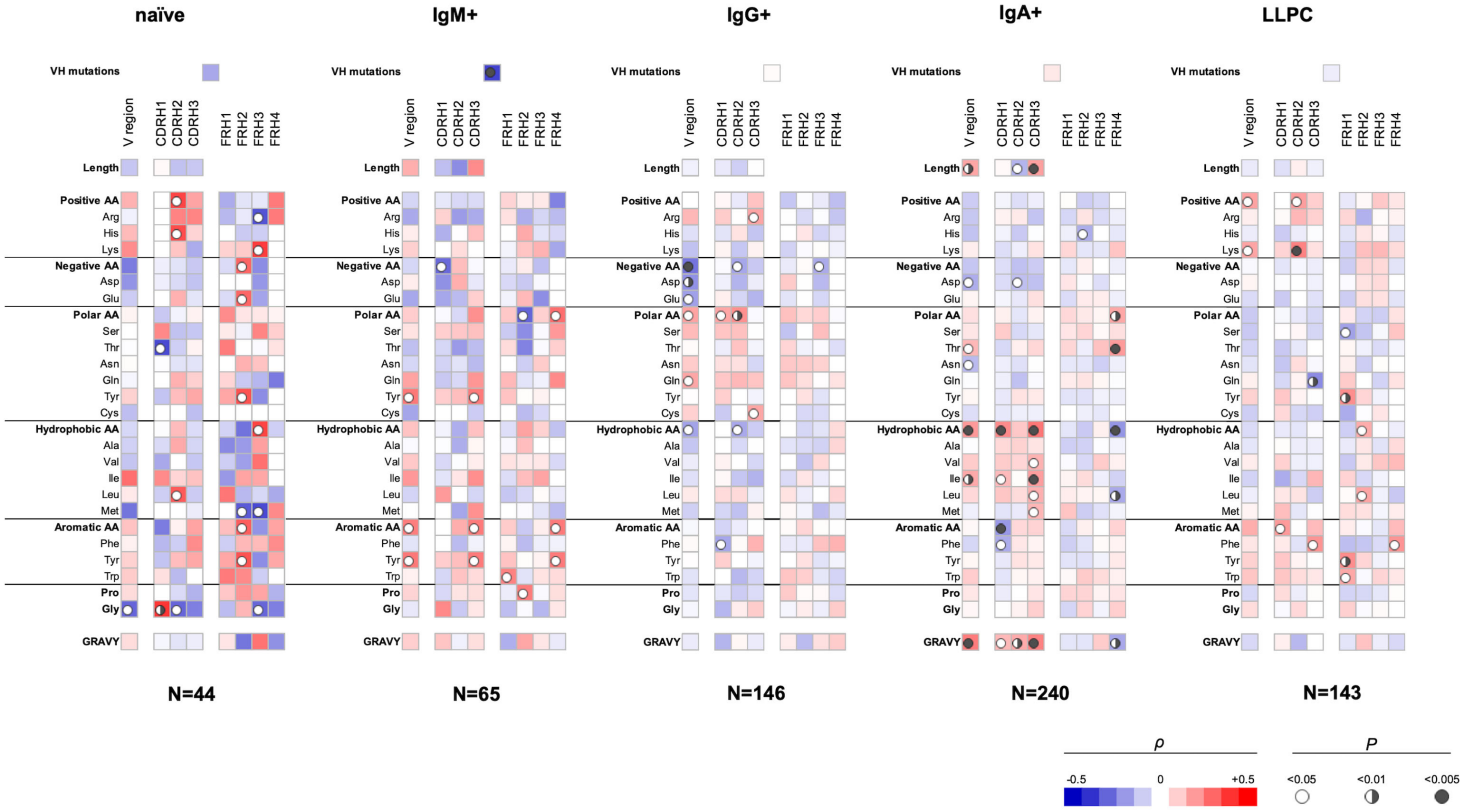
Correlation analyses of polyreactivity of human Abs cloned from different B-cell subpopulations with sequence characteristics of V_H_ domain. The heat maps present correlation coefficient (ρ) obtained by Spearman’s rank analyses of sequences characteristics of V_H_ domains (e.g., frequency of individual amino acid residues, type of residues, length of CDR H loops, mutation loads, and hydrophobicity GRAVY index) with the extent of the antigen-binding polyreactivity. Abs were grouped according to their B-cell origin naive, IgM+ memory, IgG+ memory, IgA+ memory, and long-lived plasma cells. The blue color signifies a negative correlation; the red color signifies a positive correlation. The statistical significance (p-value) is indicated by a circle. Open circle p < 0.05; semi-closed circle p < 0.01, and closed circle p < 0.005.

We applied Spearman non-parametric correlation analysis to assess the sequence patterns of V_H_ and V_L_ domains associated with polyreactivity of human Abs. The obtained data from correlation analysis for the V_H_ domain are depicted in **Figure 1**. These data demonstrated that Ab polyreactivity significantly correlates with increased or reduced frequencies of certain amino acid residues in the V domain. Strikingly, distinct patterns of significant correlations were observed between the groups of Abs originating from different B-cell subpopulations (**Figure 1**). Thus, polyreactivity negatively correlated with the number of V_H_ somatic mutations for Abs originating from IgM+ B cells, but no significant correlation was found in other groups. In addition, a positive correlation between the length of CDR H3 and polyreactivity was observed only for Abs cloned from IgA+ B cells. The polyreactivity of Abs from three B-cell subtypes—naive, IgG+ memory and plasma cells, correlated with the presence of a higher number of basic amino acid residues (Arg, His, and Lys) in CDR H2 (naive B cells and LLPC) and CDR H3 (IgG+ memory B cells) and within entire V_H_ region (LLPC). However, no increased number of basic amino acid residues in any part of the V region was associated with the polyreactivity of Abs isolated from IgM+ and IgA+ memory B cells. Reduced number of acidic amino acid residues (Asp and Glu) in the entire V_H_ region or in CDR H1 and CDR H2 was associated with a higher polyreactivity in the case of Abs cloned from all types of memory B cells but not from naive B cells and LLPC (**Figure 1**). Abs cloned from each B-cell subpopulation demonstrated unique patterns of positive or negative correlates of polyreactivity with the number of polar amino acid residues in V_H_ domain. The presence of polar residues was especially important for determining the polyreactivity of IgG+ memory B cells (**Figure 1**). Thus, a higher number of polar amino acids (as a physiochemical category) in CDR H1 and H2 and in the entire V_H_ was positively correlated with promiscuous antigen binding of Abs. Statistically significant correlations were also found for the number of Gln residues in the entire V_H_ and Cys residues in CDR H3 (**Figure 1**). Contrary, polyreactivity negatively correlated with the presence of Thr in CDR H1 in Abs cloned from naive B cells. Our data demonstrated that the polyreactivity of Abs isolated from IgA+ memory B cells significantly correlated with the presence of different hydrophobic residues (Val, Ile, Leu, and Met) in the entire V_H_ region or in CDR H1 and H3 loops (**Figure 1**). This prominent pattern was not observed in Abs isolated from other types of B cells. On the contrary, the polyreactivity of Abs from IgG+ memory B cells was negatively correlated with the number of hydrophobic amino acids (as a physiochemical category) in the entire V_H_ domain or CDR H2. The increased number of hydrophobic residues in V_H_ of Abs isolated from IgA+ B cells resulted in a significant correlation of polyreactivity with augmented hydrophobicity index (GRAVY) of all CDR loops and entire V_H_ domain (**Figure 1**). Notable patterns of sequence correlations were also observed regarding the presence of aromatic amino acid residues in V_H_ domain. Thus, the number of aromatic amino acids (as a group) or of Tyr in V_H_ or in CDR H3 positively correlated with enhanced polyreactivity of Abs cloned from IgM+ B cells (**Figure 1**). Positive correlation between polyreactivity and aromatic amino acids was also found for Abs from LLPC, but in this case, the presence of any aromatic residues in CDR H1 or Phe in CDR H3 reached significance. Notably, reverse tendencies were observed for Abs isolated from IgG+ and IgA+ B cells, where the presence of aromatic residues or Phe negatively correlated with the antigen-binding polyreactivity (**Figure 1**). The number of Gly residues significantly correlated with polyreactivity only for Abs that originated from naive B cells. Thus, a lower number of Gly in the V_H_ region and CDR H2 was associated with higher polyreactivity. However, a reverse tendency was observed for Gly residues in the case of CDR H1 (**Figure 1**).

Our data also revealed that sequence features of framework regions (FW) of V_H_ can also contribute to the polyreactivity of Abs. Importantly, we found that the patterns of significance in FW regions also depended on the origin of Abs (**Figure 1**). As FW regions are less frequently subjected to somatic mutations, the observed differences may reflect biased use of V_H_ genes in the case of Abs with extended antigen-binding polyreactivity.

**Figure 2:**
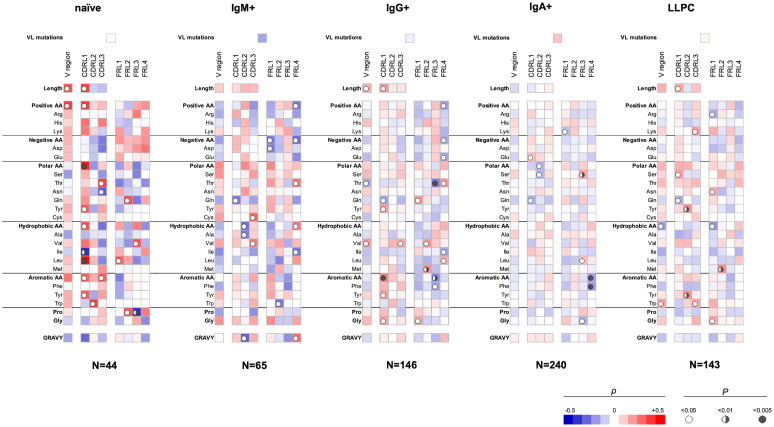
Correlation analyses of polyreactivity of human Abs cloned from different B-cell subpopulations with sequence characteristics of V_L_ domain. The heat maps present correlation coefficient (ρ) obtained by Spearman’s rank analyses of sequences characteristics of V_L_ domains (e.g., frequency of individual amino acid residues, type of residues, length of CDR L loops, mutation loads, and hydrophobicity GRAVY index) with the extent of the antigen-binding polyreactivity. Abs were grouped according to their B-cell origin naive, IgM+ memory, IgG+ memory, IgA+ memory, and long-lived plasma cells. The blue color signifies a negative correlation; the red color signifies a positive correlation. The statistical significance (p-value) is indicated by a circle. Open circle p < 0.05; semi-closed circle p < 0.01, and closed circle p < 0.005.

Our analyses showed that several sequence features of the V_L_ domain also correlate with polyreactivity of Abs (**Figure 2**). Again, different sequence patterns of correlation were detected depending on the B-cell origin of the studied Abs, albeit these differences were less prominent as compared to those observed in V_H_ (**Figures 1**, **2**). An important observation from these data is the presence of a positive correlation between the size of the entire V_L_ region or CDR L1 and polyreactive antigen binding in Abs cloned from three different B-cell subpopulations, naive, IgG+ memory, and LLPC. Although the data did not reach statistical significance, the same tendency was present in the case of IgM+ memory B cells. These data suggest that the length of CDR L1 is an important determinant of polyreactivity of Abs. Interestingly, statistical significance was present between the lower numbers of Gln residues in CDR L1 and antigen-binding polyreactivity in all B-cell subpopulations except in naive B cells (**Figure 2**). Of note, this CDR L1 is characterized by the highest variability in length among CDRs in V_L_ region. Another common sequence trait positively correlating with polyreactivity of different B-cell subpopulations is the presence of a higher number of aromatic amino acids in all CDR loops. Such correlation reached significance for Abs cloned from naive-, IgG+, and LLPC (**Figure 2**). An important general observation of the correlation analyses of V_L_ domain is that the region that has the most significant capacity to determine the promiscuous antigen binding of Abs, from different B-cell subpopulations is CDR L1 (**Figure 2**). Similarly, as the observation for V_H_, the sequence features in FW regions correlating with polyreactivity of Abs showed particularities as dependent on the B-cell type from which Abs were cloned.

Taken together, the data from Spearman correlation analyses of V_H_ and V_L_ performed on Abs categorized based on their B-cell origin unravel numerous sequence patterns significantly correlating with the antigen-binding promiscuity. Notably, unique patterns were present in different groups of Abs.

### Compatibility between Ab repertoires

It is noteworthy that the Abs cloned from IgA+ memory B cells (n=240) were analyzed for polyreactivity by ELISA (13), whereas the binding promiscuity of the rest of Abs (n=398) was assessed by PSR assay (27). We realized that the use of two distinct approaches can introduce bias in data, and direct comparison of the results should be interpreted with caution. However, previous analyses demonstrated that PSR and ELISA methods significantly correlate in their capacity to identify polyreactive Abs (21, 22). The validity of our results was also supported by the fact that there were considerable differences in the sequence patterns of V_H_ determining polyreactivity in groups of Abs that were assessed by an identical polyreactivity assay (PSR). For example, note the differences between Abs from IgG+ memory B-cell group (n=146) and Abs from LLPC (n=143) (**Figure 1**).

### Correlation analyses of sequence features of V_H_ and V_L_ regions determining the polyreactivity of Abs performed on integrated Ab repertoire

**Figure 3:**
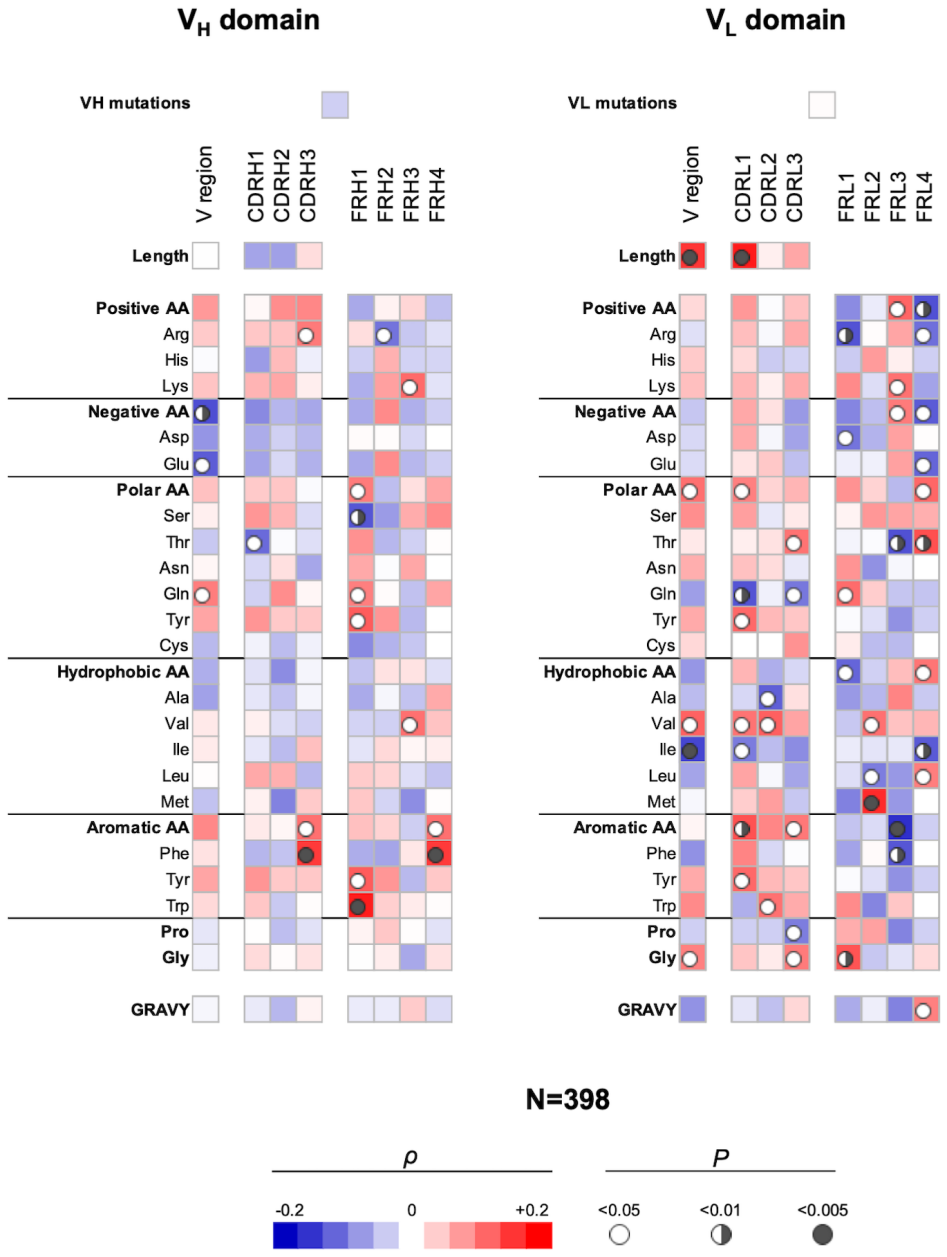
Correlation analyses of polyreactivity of human Abs cloned from different B-cell subpopulations with sequence characteristics of V_H_ and V_L_ domains. The heat maps present correlation coefficient (ρ) obtained by Spearman’s rank analyses of sequences characteristics of V_H_ (left panel) and V_L_ (right panel) domains with the extent of the antigen-binding polyreactivity. Analyses were performed on integrated Ab repertoire consisting of Abs isolated from naive, IgM+ memory, IgG+ memory, and long-lived plasma cells. The blue color signifies a negative correlation; the red color signifies a positive correlation. The statistical significance (p-value) is indicated by a circle. Open circle p < 0.05; semi-closed circle p < 0.01, and closed circle p < 0.005.

Next, we elucidated the sequence correlates of V regions that determine antigen-binding polyreactivity in an integrated set of Abs originating from different B-cell subpopulations. We excluded from this bulk repertoire only Abs originating from IgA+ B cells, as their polyreactivity was assessed by a different technique. By performing the statistical analyses of a panel of 398 Abs, a number of significant correlations were observed (**Figure 3**). Thus, Ab polyreactivity in bulk repertoire correlated with the presence of a higher number of Arg residues in CDR H3 and a lower number of acidic amino acids (especially Glu) in the entire V_H_ domain. Polyreactivity was also negatively correlated with Thr in CDR H1 and positively associated with the number of Gln residues in the whole V_H_. No significant correlation was observed between the number of hydrophobic amino acids in CDR loops of V_H_ domain and the polyreactivity of Abs. Notably, higher polyreactivity of Abs correlated with an increased number of aromatic residues (Phe and Trp) in CDR H3 (**Figure 3**). The number of aromatic residues in FR1 was also significantly elevated in Abs with higher antigen-binding polyreactivity.

The correlation analyses revealed even a larger number of significant correlations of V_L_ sequence characteristics with the polyreactivity in bulk Ab repertoire (**Figure 3**). Thus, a positive correlation was observed between the length of V_L_ region and particularly of CDR L1 and promiscuous antigen binding. In addition, the number of polar amino acids in the V_L_ or in CDR L1 and L3 correlated positively (Tyr and Thr) or negatively (Gln) with the Ab polyreactivity. The presence of the hydrophobic amino acid Val in the entire V_L_ and in CDR L1 and L2 was similarly significantly associated with increased polyreactivity of Abs. However, the presence of Ile in whole V_L_ and Ala in CDR L2 had a significantly negative impact on the Ab promiscuity (**Figure 3**). Similarly, as in the case of V_H_, polyreactivity of Abs in integrated Ab repertoire positively correlated with the presence of aromatic amino acid residues in CDR L1 (Tyr), CDR L2 (Trp), and in CDR L3 (as type). Polyreactivity of Abs also significantly correlated with an increased number of Gly residues in V_L_ as well as in CDR L3 (**Figure 3**).

Our analyses demonstrated that the FW regions of light-chain V regions have a more decisive role in determining polyreactivity in integrated Ab repertoire as compared with the FW region of heavy V regions (**Figure 3**). This observation suggests that a larger set of V_L_ genes may be associated with the polyreactivity of Abs.

Collectively, these results revealed several significant correlates of Ab polyreactivity in bulk immune repertoire consisting of Abs from distinct B-cell subpopulations. These data showed certain prominent sequence patterns associated with polyreactivity—lack of acidic residues in V_H_ and presence of aromatic amino acids in CDRs of both V_H_ and V_L_. The sequence pattern correlating with polyreactivity in bulk repertoire differed from the patterns in Abs from a particular B-cell subtype. Moreover, the strength of correlations was diminished, suggesting the presence of multiple pathways for the achievement of polyreactive antigen binding.

### Analyses of Abs manifesting the highest level of antigen-binding polyreactivity

To obtain further information about molecular correlates of polyreactivity, we next focused our analyses only on Abs demonstrating the most prominent antigen-binding promiscuity. To this end, we selected the top 5 polyreactive Abs from each B-cell subpopulation. **Table 1** presents a summary of the characteristics of the variable regions of the Abs. These data revealed that the most polyreactive Abs did not have any specific bias in the lengths of CDR loops or their isoelectric points. Thus, CDR H3 and CDR L1 regions displayed the highest variability of lengths among different polyreactive Abs, i.e., 8–26 and 6–12 residues for CDR H3 and CDR L1, respectively (**Table 1**). *In silico* calculated isoelectric points of CDRs also varied in a very broad range between different Ab molecules (**Table 1**). For example, in the case of the most diverse and important region for antigen recognition (CDR H3), the calculated pI values of the strongly polyreactive Abs were in the range between 3.67 and 9.14. It is noteworthy that substantial variability in the length of CDR loops and the pI values was observed between Abs within all groups, except in the case of Abs isolated from IgA+ B cells (**Table 1**). These data indicate that a substantial level of antigen-binding promiscuity can be achieved by alternative topological and physicochemical features of the antigen-binding sites.

**Figure 4:**
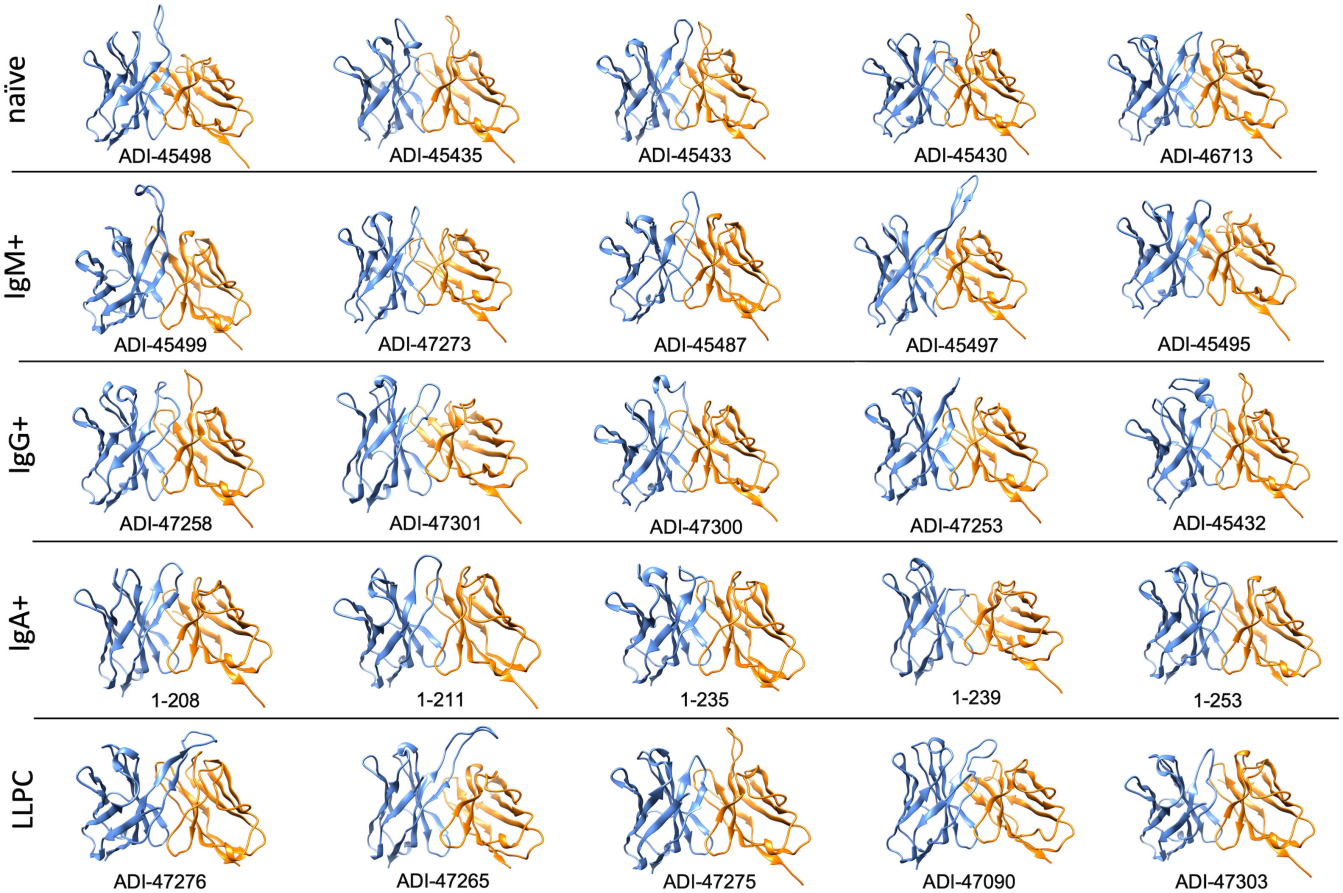
Molecular models of V regions of polyreactive Abs. Molecular models of the Abs that manifest the highest level of antigen-binding polyreactivity from each B-cell category. The Abs were ordered according to the level of polyreactivity (PSR score, except for IgA+ memory B cells group) in descending order from left to right. Side view of the V region is presented. The blue ribbon corresponds to V_H_; the orange ribbon corresponds to V_L_. The models were generated with the Rosie-2 Ab-module of the Rosetta online server and visualized by the molecular viewer UCSF Chimera software v. 1.16.

Furthermore, to gain more detailed information about the topology of the antigen-binding sites of polyreactive Abs, we applied the structural modeling algorithm Rosie-2 (Rosetta Commons). The side views of the most probable structures of V regions of Abs are shown in **Figure 4**. These models demonstrated that the highly polyreactive human Abs have diverse topologies of their variable regions. One apparent feature observed in a part of the Abs is the presence of long and protruding CDR H3 (**Figure 4**). This structural feature was especially evident in polyreactive Abs from naive B cells (ADI-45498), IgM+ memory B cells (ADI-45499, ADI-45497), and LLPC (ADI-47276 and ADI-47265) and well corresponded to the length of CDR H3 (**Table 1**). Interestingly, the structural models also indicated that some Abs have projecting only CDR L1 (ADI-45435, ADI-45430, and ADI-47275), or in some cases, both CDR H3 and CDR L1 are protruding (ADI-45435). Despite this obvious structural feature of variable regions of some of the polyreactive Abs, the structural models also showed that another fraction of highly polyreactive Abs did not display abnormally protruding CDR loops (**Figure 4**). Indeed, the topology of their binding sites is similar as the one depicted for most of the protein-binding Abs available in Ab structural databases. This observation further substantiated our results from correlation analyses, suggesting that polyreactive Abs use diverse pathways for the achievement of broad antigen-binding promiscuity.

**Table 1:** Characteristics of top polyreactive antibodies used for molecular modeling analyses.

Origin / B cell subset	Antibody	Polyreactivity score*	VH mutation number	CDRH1		CDRH2		CDRH3		VL mutation number	CDRL1		CDRL2		CDRL3	
				Length (a.a.)	Isoelectric point	Length (a.a.)	Isoelectric point	Length (a.a.)	Isoelectric point		Length (a.a.)	Isoelectric point	Length (a.a.)	Isoelectric point	Length (a.a.)	Isoelectric point
Naive	ADI-45498	0,71069496	0	8	5.641	8	5.476	19	8.110	0	8	5.955	3	3.596	11	3.604
Naive	ADI-45435	0,457177329	0	8	5.741	7	7.067	20	6.131	0	12	7.992	3	5.861	9	5.956
Naive	ADI-45433	0,364564497	0	8	5.708	8	6.054	17	7.065	0	11	7.141	3	5.697	7	7.105
Naive	ADI-45430	0,260866421	0	9	5.744	7	7.040	14	4.090	0	12	7.992	3	5.861	9	5.937
Naive	ADI-46713	0,19010429	0	8	5.620	8	5.646	12	7.850	0	7	5.760	3	5.811	9	5.866
IgM memory	ADI-45499	0,710238077	0	8	5.620	8	6.054	23	7.360	0	7	5.760	3	5.811	10	5.892
IgM memory	ADI-47273	0,263842045	3	8	5.834	7	5.745	14	3.780	6	6	5.815	3	8.073	9	5.942
IgM memory	ADI-45487	0,248689858	0	8	5.810	8	3.544	16	4.038	1	7	5.760	3	5.811	9	5.867
IgM memory	ADI-45497	0,212869931	0	8	5.741	7	7.067	22	4.640	0	6	5.650	3	5.885	9	8.247
IgM memory	ADI-45495	0,151349728	0	8	5.760	7	5.745	12	8.137	8	9	3.644	3	3.606	10	8.165
IgG memory	ADI-47258	0,309315889	11	8	7.151	8	3.746	19	4.247	9	12	7.981	3	5.861	9	8.210
IgG memory	ADI-47301	0,276256988	6	8	8.143	8	5.651	12	8.214	2	9	3.644	3	3.635	10	5.915
IgG memory	ADI-47300	0,237031082	7	8	7.142	7	5.793	19	9.139	4	6	5.714	3	8.073	9	5.930
IgG memory	ADI-47253	0,23696223	8	8	5.761	8	3.598	14	4.091	9	6	5.719	3	5.885	9	5.880
IgG memory	ADI-45432	0,222241241	16	8	3.612	7	3.625	26	4.521	5	12	7.992	3	5.861	9	5.929
IgA memory	1-208	4	15	8	5.753	8	5.683	15	6.117	13	6	4.089	3	5.885	9	6.044
IgA memory	1-211	4	15	8	3.497	7	5.868	12	8.101	15	6	6.905	3	5.775	9	8.269
IgA memory	1-235	4	7	8	5.820	8	3.617	14	6.297	6	6	8.268	3	5.885	9	5.781
IgA memory	1-239	4	12	8	5.716	8	5.879	8	8.311	4	6	5.721	3	5.811	9	3.651
IgA memory	1-253	4	13	8	5.817	8	5.886	14	6.110	3	7	5.674	3	5.885	10	5.920
LLPCs	ADI-47276	0,567042536	27	8	5.873	8	6.259	22	7.917	11	6	5.807	3	8.073	9	7.123
LLPCs	ADI-47265	0,487816941	16	8	5.834	8	5.958	23	7.808	5	7	5.663	3	5.811	10	5.947
LLPCs	ADI-47275	0,4180419	24	8	5.815	8	5.802	11	4.041	3	12	8.062	3	5.861	9	8.295
LLPCs	ADI-47090	0,24495424	15	8	4.042	8	8.256	18	3.670	7	8	3.559	3	3.569	11	3.626
LLPCs	ADI-47303	0,201395637	18	8	3.581	8	8.618	12	7.112	10	7	8.574	3	5.774	9	3.609

*For antibodies cloned from naïve-, IgM+ memory-, IgG+ memory B cells and LLPC PSR scores are displayed as published in 27. For IgA+ memory B cells the polyreactivity was assessed by ELISA and Hep-assays. A numeric score 0-4 was assigned based on the number of recognized antigens and strength of the signal.The colors in Table 1 indicate different subsets of B cells from which studied antibodies originate.

**Figure 5:**
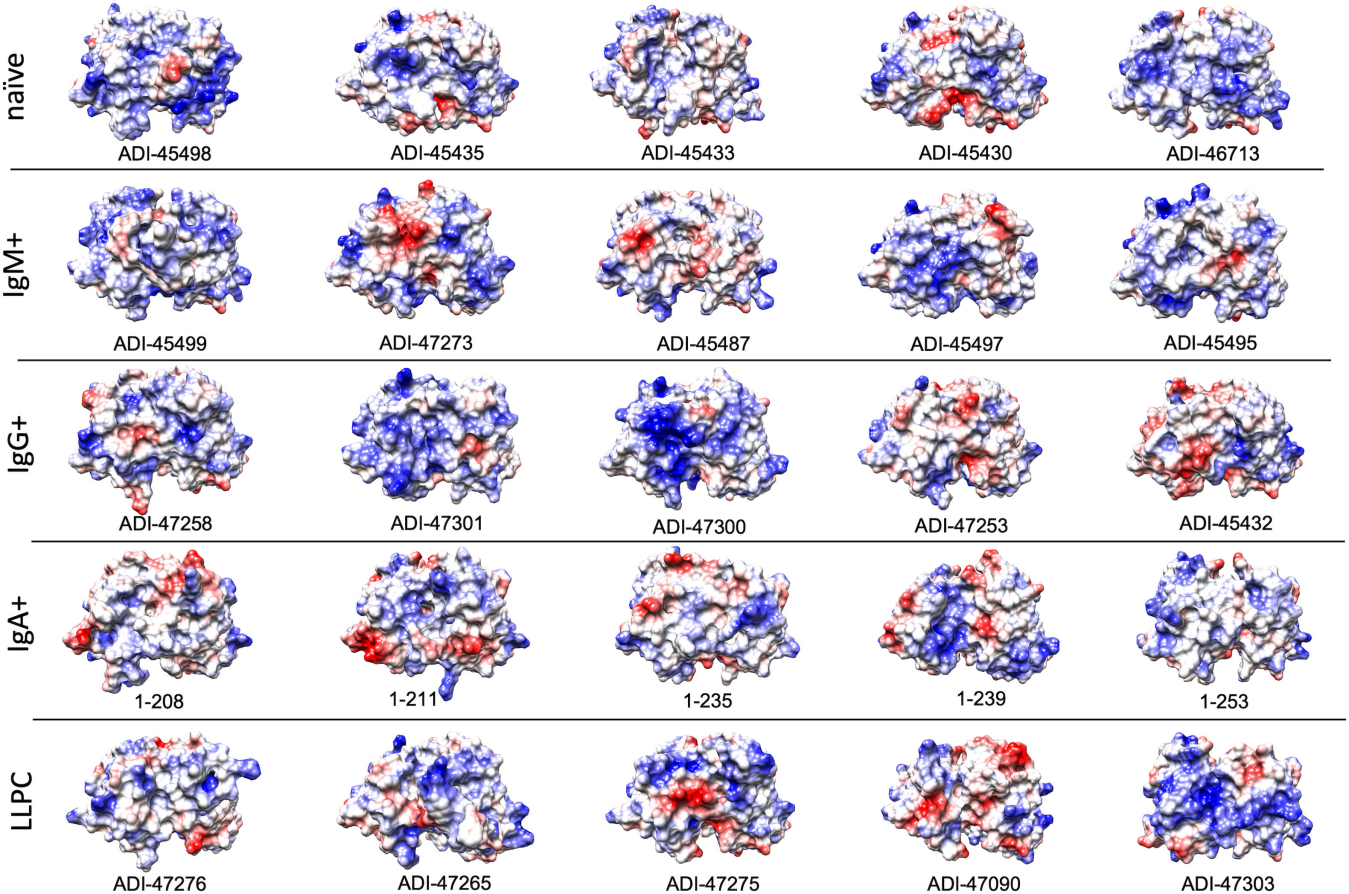
Surface electrostatic coloring of the antigen binding sites of polyreactive Abs. Top view of the structures of V regions of the most polyreactive Abs, isolated from different B-cell subtypes. The V regions are ordered in descending order of their polyreactivity (from left to right). The structural models were generated by using the Rosie-2 Ab module of the Rosetta online server. Electrostatic charges (blue, positive; red, negative) were depicted by using UCSF Chimera software v. 1.16.

**Figure 6:**
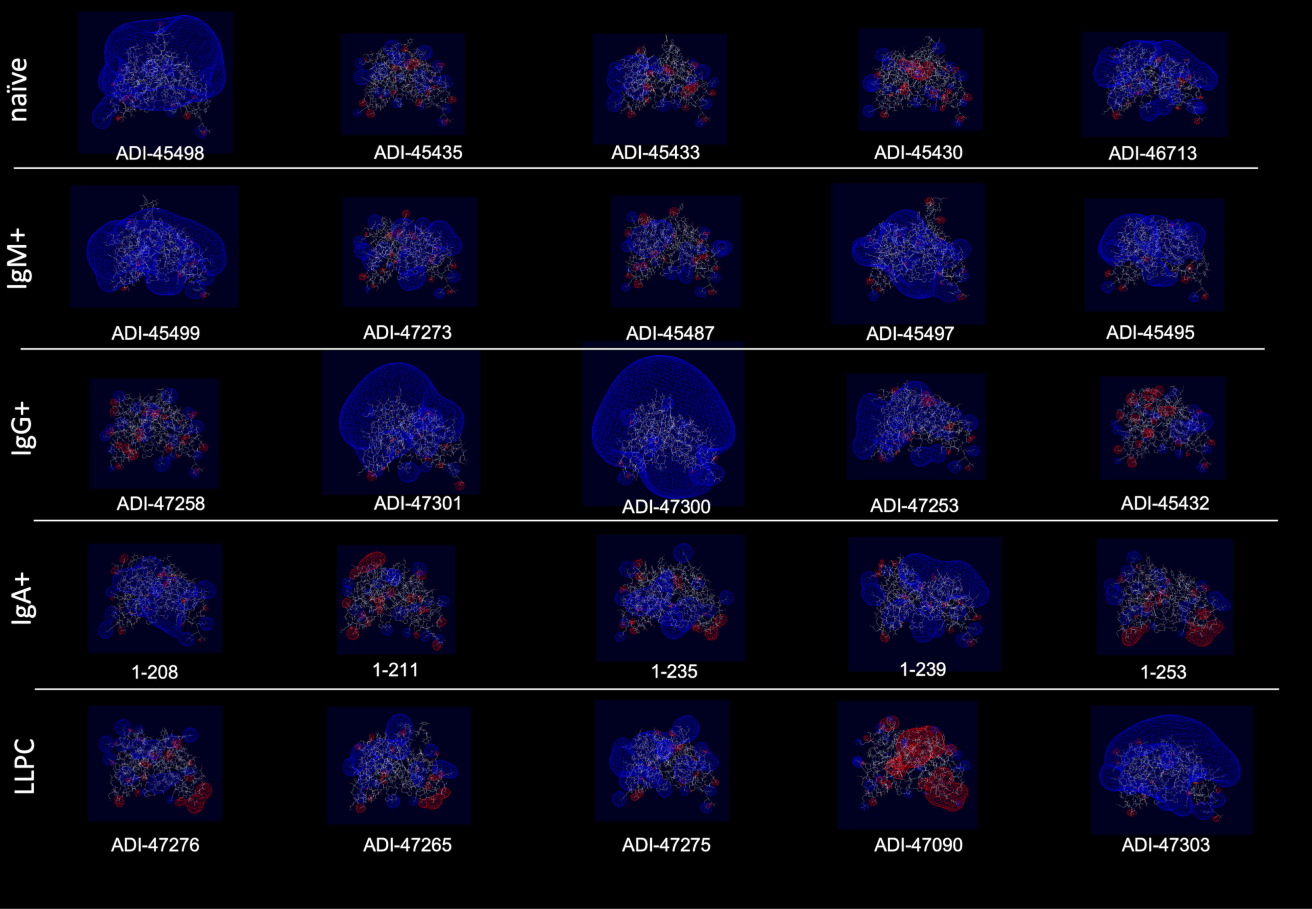
Three-dimensional electrostatic potential of V region of selected polyreactive Abs. Side view of the structural models of V regions of the most polyreactive Abs, isolated from different B-cell subtypes. The models were obtained by using the Rosie-2 Ab module of the Rosetta online server. The V regions are ordered in descending order of their polyreactivity (from left to right). The pictures show Coulombic electrostatic potentials (blue, positive; red, negative) that were calculated and visualized by using Swiss-PdbViewer v. 4.1.

Polyreactivity has been associated with specific physiochemical characteristics of antigen-binding sites of Abs such as the presence of patches of positive charges on the molecular surface. To elucidate whether this is the case in the human Abs isolated from different B-cell compartments, we depicted the distribution of charges on the surface of antigen-binding sites of a selected set of Abs characterized with outstanding polyreactivity (**Figure 5**). We also calculated three-dimensional Coulombic surface electrostatic potential of variable regions of the selected Abs (**Figure 6**). These data demonstrated that different polyreactive Abs display distinct patterns of distribution of charged or non-polar residues in their binding sites (**Figure 5**) and that the energy and space distribution of their electrostatic potentials differs substantially (**Figure 6**). Some of the most polyreactive Abs, indeed, demonstrated extensive positively charged antigen-binding sites (for example, ADI-45498, ADI-46713, ADI-45499, ADI-47301, ADI-47300, and ADI-47303); moreover, the data revealed that a predominant fraction of Abs had antigen-binding surfaces deprived of negative charges. Nonetheless, there were highly polyreactive Abs with balanced positive and negative charged patches or in some cases; Abs had antigen-binding sites that carry little charged residues or even predominantly negative charges (**Figures 5**, **6**).

**Figure 7:**
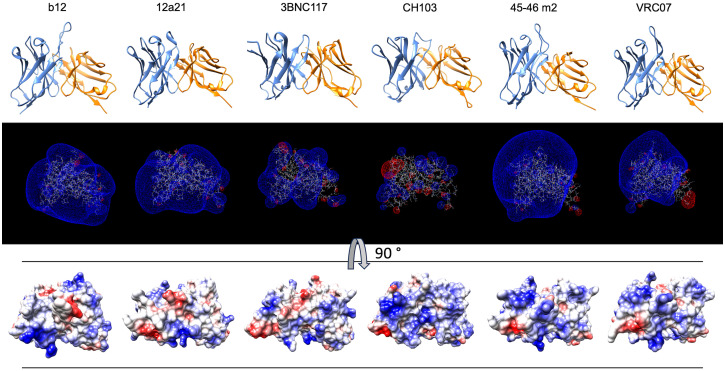
Structures and physicochemical features of a set of polyreactive HIV-1 broadly neutralizing antibodies. Structures (side view), three-dimensional electrostatic potentials (side view), and surface electrostatic coloring (top view) of V regions of polyreactive bNAbs—b12, 12a21, 3BNC117, CH103, 45-46m2, and VRC07. For visualization of the structures of V domains PDB files 2NY7, 4JPW, 4JPV, 4JAM, 4JKP, and 4OLU were used. The structures and surface distribution of the electrostatic charges (blue, positive; red, negative) were visualized by using UCSF Chimera software v. 1.16. The three-dimensional Coulombic electrostatic potentials (blue, positive; red, negative) were calculated and visualized by using Swiss-PdbViewer v. 4.1.

To confirm and broaden these observations, we further analyzed the structural and physicochemical characteristics of polyreactive Abs that bind to an identical epitope. A large number of broadly neutralizing HIV-1 Abs (bNAbs) are known to manifest antigen-binding polyreactivity (12, 13, 52). The Abs b12, 12a21, 3BNC117, CH103, 45-46m2, and VRC07 all bind to the same region on the HIV-1 envelope protein, i.e., gp120 CD4 binding site and were reported to be polyreactive (12, 13, 52, 53). Importantly, the structures of Fab regions of these Abs have been determined by X-ray analyses (54–58). Our comparative analyses of the topology and the physiochemical properties of antigen-binding sites of a set of polyreactive bNAbs demonstrated that there is not a common structural pattern that could explain their promiscuous antigen binding. Thus, b12, 45-46m2, and VRC07 have long and protruding CDR H3 loops (**Figure 7**), whereas other polyreactive bNAbs have short and less exposed CDR H3. The polyreactive bNAbs also substantially differ in their three-dimensional electrostatic potentials and in the distribution of charges in their antigen-binding sites (**Figure 7**). Thus, Abs 45-46m2 and VRC07 have predominantly positively charged antigen-binding sites with a potent positive electrostatic potential. However, the positive patches could not explain the polyreactivity of b12, 12a21, and 3BNC117, as these Abs have a more balanced distribution of the positive and the negative charges in their antigen-binding sites (**Figure 7**). These data corroborate the results from our analyses of the physicochemical properties of the *in silico* modeled structures of V regions of polyreactive Abs (**Figures 4** **–** **6**). Moreover, they indicate that even among the polyreactive Abs targeting the same region in antigen, there could be distinct molecular mechanisms for the attainment of promiscuous antigen binding.

Collectively, the data from this part of the study strongly suggest that the polyreactive Abs do not use universal topological or physiochemical qualities that determine the binding to multiple unrelated antigens. Rather, variable regions with diverse properties can endow Abs with binding promiscuity.

## Discussion

In this study, we deciphered the sequence correlates in V_H_ and V_L_ regions determining polyreactivity of human Abs. Our data show that Abs originating from different B-cell subtypes rely on distinct sequence patterns for their promiscuous antigen binding. This finding suggests that the mechanism of promiscuous antigen binding might evolve during the immune response, and it might be tailored to specific physiological roles of diverse B-cell types. Our data confirmed some previously documented sequence determinants of polyreactivity, but they also highlighted several unrecognized ones. Furthermore, data from molecular modeling of V regions of selected Abs with the highest level of polyreactivity and some polyreactive HIV-1neutralizing Abs implied the existence of alternative mechanisms that govern the antigen-binding promiscuity. Our data might explain some contradictions in the literature regarding the role of specific sequence motifs or molecular features of V regions for polyreactivity.

To assess sequence traits of V domains associated with polyreactivity, we applied non-parametric correlation analyses (Spearman’s rank order correlation) using sequence and antigen-binding data for >600 human Abs, unbiased by selection for a particular antigen specificity. The advantage of this approach is that it evaluates the individual contribution for Ab polyreactivity of every amino acid residue (or type of residues) in all parts of the V domain (CDRs and FWs). As polyreactivity/monoreactivity of Abs is not a binary measure but rather spreads as a continuum of varying extents, the correlation analyses can reveal subtle details in the sequence determinants of antigen-binding promiscuity without the need for *a priori* grouping of Abs as polyreactive and monoreactive. A key aspect of this study is that we applied the correlation analyses not only to bulk Ab repertoires but also to groups of Abs stratified based on B-cell type from which they originate: naive, IgM+ memory, IgG+ memory, IgA+ memory, and long-lived plasma cells.

Our data showed that Abs originating from various human B-cell subpopulations have different sequence determinants of their polyreactivity, especially in their V_H_ domain (**Figures 1**, **2**). From analyzing the heatmaps depicted in **Figure 1**, we can conclude that Abs rely on five major groups of sequence attributes for their polyreactivity—i) an increased number of positively charged residues in CDRs, ii) a reduced number of negatively charged residues in the entire V_H_ and CDRs, iii) an increased number of aromatic residues in V_H_ and CDRs, iv) a reduced number of aromatic residues in CDR H1, v) an increased number of polar residues in V_H_ and CDRs, and vi) an increased number of hydrophobic residues in V_H_ and CDRs. Notably, our data indicated that different categories of Abs use unique combinations of these sequence features for achieving polyreactivity, and in some cases, polyreactivity correlates with contrasting among the groups sequence characteristics. For example, polyreactive Abs cloned from naive B cells rely on a higher number of positively charged residues in their CDR H2. Similarly, higher polyreactivity of Abs cloned from LLPC correlated with the presence of an increased number of positively charged residues in CDRs. However, the polyreactivity in this group of Abs is also associated with a significantly elevated number of aromatic residues in CDR H3. On the other hand, polyreactivity of Abs from IgG+ memory B cells correlated strongly with reduced numbers of negatively charged residues, an increased number of Arg in CDR H3, and an increased frequency of polar amino acid residues in V_H_ and CDRs. In contrast to other categories, Abs from IgM+ memory B cells rely exclusively on aromatic residues for promiscuous antigen binding. Interestingly, a unique pattern was detected also for Abs from IgA+ memory B cells (**Figure 1**). Thus, polyreactivity of these Abs significantly correlated with the presence of a higher number of hydrophobic amino acid residues in V_H_ and CDRs (Leu, Ile, and Met) and an increased length of CDR H3. The polyreactivity of Abs isolated from IgA+ B cells correlates with a significantly reduced number of aromatic residues in CDR and the tendency for a lower prevalence of positively charged residues.

The V_L_ sequence characteristics that had a strong correlation with polyreactivity were the length of CDR L1 and the presence of aromatic residues in CDRs. Of note, these patterns were constantly present in V_L_ domains of Abs from all groups with exception of Abs cloned from IgM+ and IgA+ memory B cells (**Figure 2**). The elevated frequency of positively charged amino acid residues in V_L_ significantly correlated with polyreactivity only in case of Abs cloned from naive B cells. The CDR L1 region displays the highest variability in size among the three CDR L loops. One can speculate that the longer CDR loops can contribute to higher conformational dynamics, thus promoting promiscuous antigen binding.

In addition to identified determinants in CDRs, the statistical analyses uncovered important correlations of different amino acids in FWs of V_H_ and V_L_ with polyreactivity. These correlations were especially pronounced in both V domains of Abs isolated from naive and IgM+ memory B cells. The association of sequence traits of FWs in unmutated or weakly mutated V regions with polyreactivity might indicate biased usage of specific genes encoding V domains. Indeed, the contribution of some V gene segments for polyreactivity has already been demonstrated in different Ab repertoires (15, 27, 35).

A sequence attribute that has been associated with polyreactivity of Abs is the mutation load in V_H_ and V_L_ domains. However, in the literature, there is controversial evidence about the role of this attribute. Thus, some studies show that the percentage of polyreactive Abs is the highest among germ-line Abs, whereas other works demonstrated a higher prevalence of polyreactivity among Abs with mutated V regions (15, 23, 24, 27, 37, 41, 59, 60). Our analyses revealed that a low number of mutations in V_H_ significantly correlates with polyreactivity of Abs cloned from IgM+ memory B cells (**Figure 1**). In contrast, the mutations in V_H_ or V_L_ do not show a significant correlation with binding promiscuity of human Abs that were cloned from the other types of B cells.

Previous works demonstrated an association of a higher number of positively charged amino acid residues in CDRs or the presence of positively charged patches with polyreactivity of Abs (15, 23, 26–33). Our statistical analyses confirmed these reports, but they also clearly demonstrated that this is not the determinant of polyreactivity for all groups of Abs and that this is not the strongest polyreactivity determinant. Molecular modeling of electrostatic potentials (**Figures 5**, **6**) of the highest polyreactive Abs also demonstrated that positive charges are not exclusively present in all V regions. The statistical analyses demonstrated that there are two additional drivers of polyreactivity—increased prevalence of aromatic amino acids in CDRs of V_H_ and V_L_ and an increased hydrophobicity of CDRs. The former is the strongest correlate for polyreactivity present both in V_H_ and V_L_. To the best of our knowledge, in the literature, there are no systemic observations linking the higher number of aromatic amino acids or hydrophobic amino acids (as in the case of positively charged residues) as drivers of polyreactivity. There, are however, sporadic reports showing the association of Trp, Val, and Phe with polyreactivity in artificial Ab libraries (34) and in the case of some engineered monoclonal broadly neutralizing HIV-1 Abs, where the introduction of single or few aromatic residues resulted in a dramatic gain of antigen-binding promiscuity (13, 57). As a consequence of their specific physicochemical and steric properties, aromatic residues in CDR would provide a capacity of Abs to establish numerous interactions with different macromolecules (61–63). A high level of hydrophobic patches also gives a particular pattern of promiscuity through binding with similar hydrophobic regions on other macromolecules (63).

The finding that Abs originating from different B-cell subpopulations have different sequence features that determine their polyreactivity might have important biological repercussions. These mechanisms may reflect different functional requirements of binding promiscuity for Abs from different B-cell subpopulations. Thus, the fact that Abs cloned from IgA+ memory B cells use mostly hydrophobic residues as determinants of polyreactivity may reflect that these Abs operate mainly at mucosal surfaces. Indeed, the important role of Ab promiscuity of mucosal IgA has been emphasized by various recent works (7, 8, 36, 64, 65). We hypothesize that polyreactivity that rely on hydrophobicity is required for the function of mucosal IgA, as intestinal and respiratory epithelial surfaces are covered with a mucous layer that is strongly negatively charged (66). If polyreactivity of IgA was mediated by positively charged residues or patches in V regions as in other cases, these Abs would be restrained by binding to highly charged mucous and would not be able to interact with bacteria or other pathogens. The hydrophobicity of IgA may also be directing Abs for binding to hydrophobic motifs on the bacterial cell walls, preventing bacterial adhesion (67). On the other hand, the predominant role of aromatic residues for polyreactivity of IgM+ memory B cells may offer a capacity of these Abs to bind to repetitive polysaccharide epitopes, which are typical for T-cell-independent antigens. Indeed, aromatic amino acids are the main type of amino acids implicated in the recognition of glycans (68).

The length of CDR loops has been related to the polyreactivity of antibodies (23, 27, 36, 37). Indeed, a longer CDR would result in a higher probability for conformational flexibility and, as a consequence, adaptability to various epitopes that may result in autoreactivity and polyreactivity (69, 70). The visualization of V regions of the most polyreactive Abs, indeed, demonstrated that some molecules have outstandingly longer CDR loops, especially CDR H3 and CDR L1 (**Table 1** and **Figure 4**). However, many of the modeled Abs did not show longer than average CDRs. This result strongly suggests that albeit some polyreactive antibodies may use long CDRs and eventual extended structural dynamics of binding sites for achieving polyreactivity, this mechanism is not employed by all Abs able to bind multiple antigens.

In this study, we also compared structures of the V regions of a set of polyreactive HIV-1 bNAbs that were previously determined by X-ray crystallographic analyses (54–58). The selected Abs target an identical region on gp120 (CD4 binding site). The comparison of the structural features of these Abs revealed alternative patterns of charge distributions and different overall topologies of their antigen-binding sites (**Figure 7**). This observation further corroborates results from the molecular modeling of polyreactive Abs from healthy humans (**Figures 4** **–** **6**) and suggests that different physicochemical traits might determine the polyreactivity of Abs, even of those Abs binding with high affinities to an identical region in the antigen.

Finally, our study rises an important question of whether Abs using different molecular mechanisms of polyreactivity would display different biological activities. It is plausible that Abs that participate in the first line of defense against pathogens and the ones that contribute to the clearance of damaged cells employ distinct molecular mechanisms for their polyreactivity. Moreover, it remains unknown whether the negative impact of polyreactivity on therapeutic monoclonal Abs differs depending on the underlying molecular mechanism. To address these questions, appropriate animal models should be implemented where immune defense, anti-inflammatory, and pharmacokinetics of different types of polyreactive Abs should be compared.

In conclusion, this study provides evidence for the implications of different molecular mechanisms in the polyreactivity of human Abs. It also demonstrates that the sequence features that determine the polyreactivity of Abs depend strongly on the B-cell origin of Abs. Thus, the study contributes valuable information about polyreactivity during the evolution of the humoral immune response. Moreover, it reveals a number of important correlates of antigen-binding promiscuity that would be of use for the assessment of developability or engineering of therapeutic Abs.

## Methods

### Antibody repertoires and correlation analyses

We used data sets from two studies (27, 37) where polyreactivity of human monoclonal Abs was assessed. The study of Shehata et al. published the sequence information of all monoclonal Abs. The sequence information for Abs in the study of Prigent et al. was kindly provided by Dr. Hugo Mouquet (Institute Pasteur, Paris, France). Since the polyreactivity in the study of Prigent et al. was displayed in semi-quantitative value, we assigned numerical values based on the number of recognized antigens from the ELISA panel. Since the polyreactivity in the study of Prigent et al. was displayed in a semi-quantitative value, we assigned a polyreactivity score as based on the number of antigens for which each antibody was considered as reactive by authors (score ranking from 0 to 4 antigens)

The identification of CDR regions and assessment of the number of amino acid replacements in V domains was performed by IMGT/V-Quest alignment software (https://www.imgt.org/). The GRAVY index was determined with the GRAVY Calculator (https://www.gravy-calculator.de).The polyreactivity of Abs was correlated with different features of variable regions—the number of somatic mutations in V_H_ and V_L_ domains; the length of CDR loops; the number of charged, polar, aromatic, and hydrophobic residues in the CDR and FW regions; and the frequency of individual amino acid residues in the CDR and FW regions. The correlation analyses were performed by non-parametric Spearman’s rank-order analysis using GraphPad Prism v.10 software (La Jolla, CA). A significant correlation was considered only with *p* value ≤ 0.05.

### Modeling of V regions of top polyreactive Abs and calculation of Coulombic electrostatic potentials

For modeling structures of the V regions of selected Abs that manifested the highest level of antigen-binding polyreactivity, we applied Ab structure modeling algorithm—RosettaAntibody3 (71–74). The sequences of V regions were submitted to the Rossie-2 module of the Rosetta online server (https://rosie.rosettacommons.org/). Relaxed three-dimensional models with lowest energy of coupled V_H_ and V_L_ domains were visualized by using the Chimera UCSF Chimera package. The same software was used for coloring the electrostatic charges of the molecular surfaces of the antibodies. Chimera is developed by the Resource for Biocomputing, Visualization, and Informatics at the University of California, San Francisco (supported by NIGMS P41-GM103311) (75). The three-dimensional Coulombic electrostatic potential was calculated and visualized by using Swiss-PdbViewer v. 4.1, http://www.expasy.org/spdbv/ (76).

Structural coordinates of Fab fragments determined by X-ray crystallography were obtained from the protein data bank (https://www.rcsb.org/) for the following polyreactive HIV-1 bNAbs: b12, 12a21, 3BNC117, CH103, 45-46m2, and VRC07 (PDB files: 2NY7, 4JPW, 4JPV, 4JAM, 4JKP, and 4OLU, respectively). The structural configuration and the electrostatic properties of V regions of these Abs were visualized with the same software packages as indicated before.

## Data availability statement

The original contributions presented in the study are included in the article/supplementary material. Further inquiries can be directed to the corresponding author.

## Author contributions

ML: Data curation, Formal Analysis, Investigation, Methodology, Visualization, Writing – original draft. RL: Data curation, Formal Analysis, Visualization, Investigation, Writing – original draft. JD: Conceptualization, Formal Analysis, Funding acquisition, Methodology, Supervision, Validation, Visualization, Writing – original draft.
